# Mobile Charging Strategy for Wireless Rechargeable Sensor Networks

**DOI:** 10.3390/s22010359

**Published:** 2022-01-04

**Authors:** Tzung-Shi Chen, Jen-Jee Chen, Xiang-You Gao, Tzung-Cheng Chen

**Affiliations:** 1 Department of Computer Science and Information Engineering, National University of Tainan, Tainan 700301, Taiwan; a0929256060@gmail.com; 2 College of Artificial Intelligence, National Yang Ming Chiao Tung University, Hsinchu City 300093, Taiwan; jenjee@nycu.edu.tw; 3 Department of Aerospace and Systems Engineering, Feng Chia University, Taichung 407802, Taiwan; tzuncchen@fcu.edu.tw

**Keywords:** energy efficiency, mobile charging robot, traveling salesman problem, Voronoi diagram, wireless rechargeable sensor networks

## Abstract

In a wireless sensor network, the sensing and data transmission for sensors will cause energy depletion, which will lead to the inability to complete the tasks. To solve this problem, wireless rechargeable sensor networks (WRSNs) have been developed to extend the lifetime of the entire network. In WRSNs, a mobile charging robot (*MR*) is responsible for wireless charging each sensor battery and collecting sensory data from the sensor simultaneously. Thereby, *MR* needs to traverse along a designed path for all sensors in the WRSNs. In this paper, dual-side charging strategies are proposed for *MR* traversal planning, which minimize the *MR* traversal path length, energy consumption, and completion time. Based on *MR* dual-side charging, neighboring sensors in both sides of a designated path can be wirelessly charged by *MR* and sensory data sent to *MR* simultaneously. The constructed path is based on the power diagram according to the remaining power of sensors and distances among sensors in a WRSN. While the power diagram is built, charging strategies with dual-side charging capability are determined accordingly. In addition, a clustering-based approach is proposed to improve minimizing *MR* moving total distance, saving charging energy and total completion time in a round. Moreover, integrated strategies that apply a clustering-based approach on the dual-side charging strategies are presented in WRSNs. The simulation results show that, no matter with or without clustering, the performances of proposed strategies outperform the baseline strategies in three respects, energy saving, total distance reduced, and completion time reduced for *MR* in WSRNs.

## 1. Introduction

With the technological progress of the Internet of things, the deployment of sensors has also increased demand widely in many fields, such as used in smart city, industry, precision agriculture, and so on. At present, the development of sensors has the trend toward low-power consumption of wireless communications and computation. In recent decades, researchers have proposed various powerful strategies to extend the operating time of sensors, such as wireless recharging strategies [1,2], reducing sensor consumption techniques, and harvesting energy from the environment, i.e., solar energy [3,4]. However, power converting from the source of external energy, such as solar energy, into electrical energy is unreliable due to the inefficiency of the power conversion and uncertainty of the environment. In addition, by reducing the energy consumption for sensors, the network lifetime can be extended, but the sensors energy cannot be prevented from being depleted in a long time. Thus, to gain better performance, the wireless recharging strategies to supply the sensors power for the wireless rechargeable sensor network (WRSN) [5,6] are recommended.

In a wireless rechargeable sensor network, all of the sensors are regarded as rechargeable sensors [7,8]. Meanwhile, a mobile charging robot (*MR* for short) is responsible for charging the multiple sensors by wireless power transfer technology and collecting data simultaneously. *MR* held the power by an attached rechargeable battery will keep moving towards these sensors within a limited range. Sensors are recharged over the air by dedicated wireless power transmitters for communications by *MR* [8]. By recharging from *MR*, all sensors can be extended the lifetime of the entire WRSN effectively [9]. Wireless rechargeable sensors are studied and presented by the works of Wireless Identification and Sensing Platform (WIPS) [8] and wireless powered communication networks [7]. During recharging sensors by *MR*, sensory data collection from sensors can also be conducted at the same time [7].

Sensors are deployed either randomly or prepensely in the sensing field. While monitoring and sensing the environment for a long period of time is necessary, the sensors need to care about their energy consumption. One-hop communication from each sensor to *MR* can be useful to extend their lifetime. On the other hand, both energy recharging and sensory data transmitting can be done by means of *MR* visiting [10,11]. Each sensor can obtain energy from *MR* and transfer sensory data to *MR* for storage. Thus, *MR* can be regarded as a mobile data collector and also a mobile charger. Furthermore, using wireless charging robots for energy replenishment and data collection can reduce the equipment damage rate and prevent people from working in hazardous areas. Users only need to wait for the mobile charging robot to complete charging and obtain sensory data in the designated safe area.

Path planning for *MR* is important to recharge the energy and collect the sensory data in WRSNs. There are many studies [5,12,13] showing how to supply energy for sensors by utilizing a mobile charging robot. The distance between sensor and the mobile charging robot is the key that associates with the efficiency of *MR* wireless charging. While the sensor is farther away from the *MR*, the worse the charging efficiency, the longer time it takes to refill the battery. On the contrary, the closer the distance, the better the recharging efficiency and the less the charging time it takes. For data transmission between sensor and *MR*, the distance also plays the same scenario as mentioned above. In [8,14], besides the energy spent on transmission will increase with the distance between sensor and *MR*, The moving speed of *MR* will also affect the charging efficiency. Liu et al. [14] proposed a strategy to establish a relay point to increase the charging efficiency of the mobile charging robot. Gong et al. [15] mentioned that, as MR moving speed changes, the charging benefit of the mobile charging robot to the sensor would change accordingly. Therefore, it is important for developing path planning charging strategies to consider how to maximize the energy charging efficiency, and reduce the total length of the walking path and total energy consumption of *MR* in WRSNs.

In this paper, the dual-side mobile charging strategies are proposed for *MR* to reduce the energy consumptions based on the three facts, *MR* moving, *MR* data collecting, and *MR* recharging to sensors. In the beginning, locations and residual energy of each sensor were known in advance to *MR*. Based on the information, a power Voronoi diagram is defined and constructed by *MR*. According to these power cells in the established power Voronoi diagram, two path planning charging strategies are proposed for MR charging and sensory data collecting for each sensor. The major contributions of the proposed path planning charging strategies are described as follows. While *MR* moving, recharging the battery and receiving the sensory data for two neighboring sensors can work simultaneously. While *MR* recharges two neighboring sensors along the designated path, balancing charging time can be achieved, i.e., minimizing the charging time for the two sensors is obtained. For any distribution and scale of sensor network, the path planning charging strategies can be worked well. Consequently, the total energy consumption of *MR* moving along the planned path can be minimized at a round in a WRSN.

The rest of the paper is organized as follows. Section 2 describes the related literature, mainly introducing related research on path planning of mobile charging robots. In Section 3, assumptions and problem formation are described. Section 4 will address the path planning algorithms for the mobile charging robot. In Section 5, experimental results will be analyzed and discussed. Section 6 concludes the summary of this paper.

## 2. Related Work

There are many studies proposed for recharging energy methodologies in wireless rechargeable sensors [7,16,17,18]. Hereby, these studies can be classified into three categories of recharging strategies: the TSP-like strategies, the strategies of finding rendezvous points in the sensing field, and the strategies of finding the planned stationary routes for mobile chargers. The advantages and disadvantages of three categories can be described below.

The first category is of the TSP-like strategies. At first, all of sensors information, such as locations or residual energy for all sensors, can be acquired in advance. Then, these strategies tried to find a TSP-like traversal path for visiting all of the sensors at once based on the information of location and residual energy for each sensor. Finally, the *MR* can move toward designated sensor locations to wireless charge the sensor along the planned path.

Fan, Jie, and Yujie proposed a wireless charging scheme with the optimal traveling path in WRSNs [19]. In [20], Lin, Zhou, Song, Yu, and Wu proposed an optimal path planning charging scheme, namely OPPC, for the on-demand charging architecture. The schedulability of a charging mission was evaluated in order to make charging scheduling predictable. In [21], Lin, Sun, Wang, Chen, Xu, and Wu proposed a Double Warning thresholds with Double Preemption (DWDP) charging scheme for adjusting charging priorities of different sensors to support preemptive scheduling. In [11], Mo, Kritikakou, and He addressed multiple mobile chargers coordination problems under multiple system requirements based on TSP problem. In [22], Nguyen, Liu, and Pham addressed the problem of scheduling limited mobile devices for energy replenishment and data collection in WRSNs with multiple sinks. In [23], Xu, Liang, and Jia discussed the use of a mobile charger to wirelessly charge sensors in a rechargeable sensor network so that the sum of sensor lifetimes is maximized while the travel distance of the mobile charger is minimized. In [24,25,26,27], the research problem of on-demand TSP mobile charging was addressed while sensors running out of energy were changed dynamically.

This kind of the first category is simple and suitable for any distribution and different scale of the sensor networks. The major drawback is that the length of the planned path is too long. This incurred that the time and moving consumption energy of *MR* traverse along the path. However, the charging consumption energy is less because the charging began while *MR* had arrived at the sensor locations, thus leading to less charging time.

The second category is the strategies of finding rendezvous points in the sensing field. At first, all of sensors information, such as locations or residual energy for all sensors, can be acquired in advance. Then, these strategies tried to analyze sensor information for finding a set of rendezvous points. According to the set of rendezvous points, a TSP-like traversal path for visiting all of the of rendezvous points at once. When *MR* visits a rendezvous point, it can recharge all of sensors in the wireless recharging range. Finally, the *MR* will move to each designated location of the rendezvous point, and wirelessly charge the sensors along the planned path.

In [19], Fan, Jie, and Yujie also proposed a wireless charging scheme with optimal traveling path in WRSNs in terms of finding rendezvous points named docking sites. Liu, Nguyen, Pham, and Lin proposed the grid-based algorithm, the dominating-set-based algorithm, and the circle-intersection-based algorithm are proposed to find a set of anchor points; then, the mobile charger was moved to charge sensors according to TSP path constructed based on these anchor points [9]. In [28], Zhao et al. studied how to schedule charging and allocate charging time simultaneously in WRSNs for the purpose of prolonging network lifetime and improve charging efficiency. Wang, Li, Ye, and Yang solved the recharge scheduling problem with considering the balance between energy consumption and latency; one dedicated data gathering vehicle and multiple charging vehicles were employed [29]. A novel clustering approach joining the mobile vehicles and unmanned aerial vehicles was proposed to prolong the network lifetime and reduce data overflow proposed in [30].

This second category is of complex and suitable for any distribution and different scale of sensor networks. The length of the traversal paths is less than the length of the TSP-like path presented in the strategies of the first category. However, the different charging time and charging power occurred due to different charging range while *MR* visited at some rendezvous point. It may incur more charging time and charging power consumption.

The last one is of the strategies of finding the planned stationary routes for mobile charger. At first, all of sensors information, such as locations or residual energy for all sensors, can be acquired in advance. Then, these strategies tried to analyze sensor information for finding a path of stationary route with different kinds of patterns, such as concentric circles, grid, and fixed routes. According to the planned routes, finally, the *MR* can move to designated routes to wirelessly charge the sensor along the planned path. When *MR* visited along the route, *MR* can recharge all of sensors in the wireless recharging range.

In [14], Liu, Lam, Han, and Chen proposed the joint data gathering and energy harvesting scheme in RWSNs in which the sensor nodes harvested energy from a mobile sink that moves along a pre-defined path. Wang, Yang, and Li addressed a network architecture in which the SenCar follows a two-dimensional symmetric random walk to recharge sensors in real-time [31]. In [13], Zhang et.al presented the itinerary selection and charging association scheme with a set of rechargeable devices and a set of candidates charging itineraries. The itinerary selections and corresponding charging association scheme determinations are to minimize the amount of energy, so that every device gets its required energy. The research work in [32] proposed a novel WRSN model equipped with one wireless charging drone with a constrained flight distance coupled with several wireless charging grids.

This kind of the last category is of simple and easy to find the stationary patterns for *MR* routing. The major weakness is that it hardly schedules dynamically for arbitrary distribution and density of sensors in the sensing field, due to the information of geographical distribution of sensors is out of consideration for path planning. Occasionally, parts of the paths are neglected when *MR* moving. Meanwhile, the total length of path is too long to incur larger the moving time and consumed energy of *MR*. Moreover, different residual energies and wireless charging distances exist in these sensors. It may incur lots of charging time and charging power consumption.

For developing *MR* wireless recharging strategies, the following facts need to be taking into consideration.

- Wireless charging technique is provided to charge rechargeable sensors in different ranges. The charging can be done with different distances between *MR* and rechargeable sensors. That is, the length of the acquired traversal paths from the strategies of the first category is too long.
- The larger the distance between *MR* and sensor, the more *MR* power for recharging consumed. The rendezvous points mentioned in the second category consider nothing about distantness from the *MR*, thus more power will be consumed for distant recharging.
- Adaptive path planning can be suitable for different scale of sensors and sensor distributions. The fixed patterns as in the strategies of the third category can incur extra power consumptions of *MR* moving for different scales of sensors and sensor distributions.
- Recharging of stationary points for *MR* was wasted charging time. Sensors can be recharged while *MR* moves along path to shorten the total recharging time.

Therefore, according to the above analysis, it is necessary to achieve the minimum power consumption, including the energy consumption of the *MR* moving, charging sensors when MR is moving, and collecting data from sensors. In addition, it is also considered that the location of each sensor is known in advance, but there is no need to know the remaining energy of each sensor in advance. As long as the *MR* moves nearby at that time, it can communicate with the neighboring sensors and then through the data request. The remaining energy of the neighboring sensors can be acquired. Therefore, the path planning strategies are proposed with the following considerations.

- Recharging sensor and collecting data from sensor while *MR* moving: it can improve and solve the problem of charging while *MR* moving and reduce the total charging time for the strategies of the first and second categories.
- The sensor does not need to be far away from the *MR* when charging: it can improve the problem that sensors may be too far away, reducing the total charging time and charging energy consumption for the strategies of second and third categories.
- The path will dynamically be adjusted according to the positions of sensors and the remaining energy; it can improve the problem of the strategies of third category with fixed patterns, be suitable for the environment of different sensors distribution, and reduce the energy consumption of mobile and charging.

As a result, to minimize the power consumption for *MR*, our objective tries to combine all of advantages from those mentioned in the above three categories. In this paper, our proposed strategies will only compare the strategy of the first category in experiments. As our knowledge, the proposed strategies are the first one to consider *MR* charging dual-side neighboring sensors being balanced.

## 3. Assumptions and Problem Formulation

In this section, we will first list the assumptions of this research problem and then formulate the problem to be solved in this paper.

First of all, assumptions of this paper are listed in below.

- There are *n* rechargeable sensors *s_i_* in $S=\left\{s_{i}\left|i=1,2,\ldots ,n\right.\right\}$ and a mobile charging robot *MR* in the environment.
- The deployment of sensors in the environment is dense.
- With power control, the charging range of the sensor is adjustable [16].
- The mobile charging robot has the location information of each sensor in advance and the remaining battery capacity of each sensor either can be acquired in advance or requested from each sensor while *MR* visits it in the near range.
- Different sensors have different battery capacities.
- The mobile charging robot has enough memory and power, and has the ability to charge sensors wirelessly (devices that emit wireless power); it has the ability to wirelessly charge neighboring sensors at the same time [7].
- Assuming there are no obstacles in the sensing environment, i.e., obstacle-free area.
- The sensors in the network are all stationary sensors.
- Mobile charging robots can send and receive data and charge multiple sensors at the same time.
- The mobile charging robot is on the path to ensure that it can charge the sensor and that the charging range is within the recharging radius of the sensor.

In the following, the problem of minimizing the power consumption for wireless rechargeable sensors via an *MR* will be formulated. A traversal path for wirelessly charging to all sensors will be determined in order to minimize power consumption.

**Figure 1:**
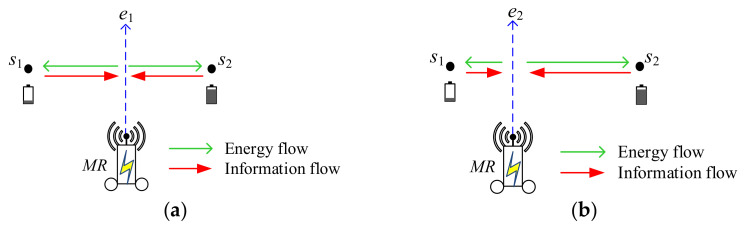
*MR* moved along different paths. (**a**) *MR* moving between two sensors, (**b**) *MR* moving toward close to sensor *S*_1_.

As in Figure 1a, *MR* moves along the perpendicular bisector, path *e*_1_, between *s*_1_ and *s*_2_. During *MR* moving, it can wirelessly charge the batteries of two sensors *s*_1_ and *s*_2_, simultaneously, as green arrows did [7]. Additionally, *MR* can receive the sensory data from sensors *s*_1_ and *s*_2_, simultaneously, as red arrows did [7,33]. However, we know that the residual energies for two sensors are different, residual energy of *s*_2_ is larger than that of *s*_1_. The recharging efficiency for *s*_2_ is better; the recharging efficiency for *s*_1_ is poor. As a result, while recharging the battery of *s*_1_, more power will draw from *MR*. Transmission of sensory data is the same situation. Suppose the *MR* is moving along the vertical line, path *e*_2_, between *s*_1_ and *s*_2_ as shown in Figure 1b, *MR* is closer to *s*_1_ than *s*_2_ during *MR* moving. Since *s*_2_ is far away from *MR*, the charging efficiency is poor. The remaining power is more and the recharging is less. Thus, *s*_1_ and *s*_2_ will be fully recharged for the total recharging time spent. This incurs that recharging efficiency and energy supply can be balanced between two sensors.

After considering whether the charging is balanced, the time duration while *MR* moving on the path also affects the sensor recharging efficiency. Faster moving speed will result in shorter moving time in case of charging time is not enough to make the battery full. In order to match the time duration of moving and recharging, the moving speed of *MR* needs to be considered for adjustment to achieve the completion of movement on the path and recharging for neighboring sensors.

As in [20], the wireless charging model *P_r_*(*d*), *d* is the distance between *MR* and sensor is defined as in Equation (1),

(1) $$P_{r}\left(d\right)=\frac{G_{s}G_{r}\eta }{L_{p}}{\left(\frac{\lambda }{4\pi \left(d+\beta \right)}\right)}^{2}P_{t}.$$

In Equation (1), $P_{t}$ denotes the source power equipped on *MR*. $G_{s}$ and $G_{r}$ denote the source antenna gain and receiver antenna gain, respectively. *λ* refers to wavelength. $L_{p}$ indicates the polarization loss. *η* represents the rectifier efficiency. The parameter *β* is assigned to be 30 [12].

**Figure 2:**
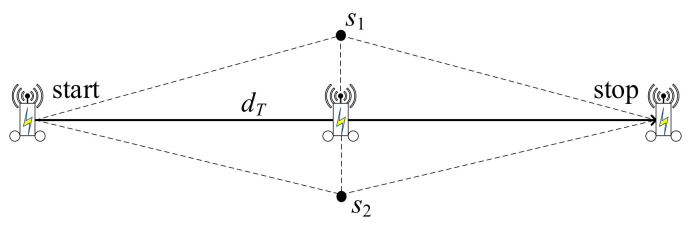
Charging efficiency of the sensor at every time point.

While *MR* is moving to recharge sensors, the charging efficiency of the sensors will be different at each time point [7]. As shown in Figure 2, the path will be affected by the *MR* moving speed. At each time point, the different *MR* locations will incur different benefits for sensors charging. In Figure 2, *MR* moving from start to stop locations with total distance $d_{T}$ is wirelessly charging the batteries of sensors *s*_1_ and *s*_2_, continuously.

It is necessary to measure the time period for charging the battery of sensor *s* with demanded power *w* when *MR* moving along the designated path with length $d_{T}$. The demanded power is defined as in Equation (2).

(2) $$w=\int_{0}^{T}P_{r}\left(dist\left(MR,s\right)\right)dt,$$

*dist*(*MR*, *s*) is defined as the distance between the locations of *MR* and *s*. *T* is time for charging from *MR* to *s*. The travel time $T=\frac{w}{I}$ with current *I* can be obtained. Thus, the moving speed is set to $V=\frac{d_{T}}{T}$. The output of the *MR* charging power is fixed, but affected by the distance. If the power demand *w* is needed, the travel time of the path is extended to achieve the effect of complete charging while traveling was completed.

In this paper, the *MR* moving speed according to the amount of charging time will be determined. When *MR* moving is completed, recharging sensors will also be completed. As in Equation (1), $P_{r}\left(d\right)$ is inversely proportional to the square of the distance *d*. Here power diagram for sensors is constructed. The distance between two neighboring power cells is inversely proportional to the square of the distance *d*. That is, when charging the neighboring sensors, the charging power can be evenly distributed to them based on their residual power energy.

Our objective is to maximize the charging efficiency, i.e., maximizing the energy usage effectiveness for wirelessly charging sensors. Since energy expenditure is composed of two parts, traveling cost and charging cost, our objective can be converted into minimizing the cost of traveling and the cost of recharging so as to maximize the charging efficiency, which is formulated as follows.

(3) $$\underset{\forall traversal\;paths}{min}\left(\overset{m}{\underset{i=1}{\sum }}eng\left(p_{i}\right)+\overset{n}{\underset{i=1}{\sum }}\left(eng_{c_{i}}+eng\left(t_{i}\right)\right)\right)$$

subject to:

(4) $$\overset{n}{\underset{i=1}{\sum }}x_{i}=n$$

(5) $$eng_{c_{i}}\geq eng_{full}-w_{i}$$

(6) $$x_{i}\in \left\{0,1\right\},\forall i\in 1,2,\ldots ,n$$

*eng*(*p_j_*) denotes the energy power consumption for *MR* moving alone *p_j_*, which is one of paths for *MR* moving, 1 ≤ *j* ≤ *k*. *eng_ci_* and *eng*(*t_i_*) denote the energy power consumptions for *MR* charging cost and information transmission cost for *s_i_*, respectively, where *t_i_* is the packet size for *s_i_* sent to *MR* [34], 1 ≤ *i* ≤ *n*. $x_{i}=1$, if ∃ *s_i_* in wireless charging range of *p_j_*, whereas $x_{i}=0$, if ∄ *s_i_* in wireless charging range of *p_j_*, for 1 ≤ *j* ≤ *m*. *w_i_* is the residual cost for *s_i_*. *eng_full_* is the full energy of each sensor.

The difficulty of this paper as noted in Equation (3) is the simple version of travelling salesman problem (TSP) for *MR* travelling to visit all of sensors. Thus, it is *NP*-*hard*. Nevertheless, the goal of this paper is to propose novel energy-efficient heuristic mobile charging strategies for wireless rechargeable sensors.

## 4. Path Planning Strategies for Mobile Charger

In this section, we will introduce the path planning of the *MR* based on the power Voronoi diagram for the WRSNs. Two algorithms are proposed for path planning to wirelessly recharge the sensors. At first, a path planning strategy based on Delaunay triangulation will be presented in Section 4.1. Next, the other path planning strategy is addressed based on dominating set for Voronoi cells on the power Voronoi diagram in Section 4.2.

### 4.1. Path Planning Strategy Based on Delaunay Triangulation

In this subsection, a path planning strategy will be proposed based on the power Voronoi diagram for all sensors. First of all, the power Voronoi diagram based on the residual energies for different sensors is established. Next, based on the constructed power Voronoi diagram, the corresponding Delaunay triangulation will be constructed. Finally, the constructed path is planed according to the triangles in the Delaunay triangulation.

In the path planning algorithm for mobile charging robots based on Delaunay triangulation (for short DT strategy), the path is perpendicular to either side of each triangle. The *MR*, passing through the designated path, can wirelessly recharge both side sensors to refill their energy with fully charged.

At first, a power diagram for sensors will be constructed. Power diagram [35] is a weighted Voronoi diagram. Here, sensors stand for the corresponding points in the power diagram. Each sensor *s_i_* will have a weighted range *r_i_* defined as $r_{i}=r\times w_{i}$ where *r* is a constant and *w_i_* is a weight, 1 ≤ *i* ≤ *n*, respectively. The power distance $d_{p}\left(s_{i},p\right)$ from any point $p\in R^{2}$ on the 2D plane to the sensor *s_i_* is defined as follow,

(7) $$d_{p}\left(s_{i},p\right)={\left\|s_{i}-p\right\|}^{2}-r_{i}^{2},\;1\leq i\leq n$$

Thus, the power Voronoi cell $vs_{i}=P\left(s_{i}\right)$ for each sensor *s_i_* is defined as in Equation (8).

(8) $$P\left(s_{i}\right)=\left\{p\in R^{2}|d_{p}\left(s_{i},p\right)\leq d_{p}\left(s_{j},p\right),i\neq j,j=1,\ldots \ldots ,n\right\}$$

By constructing a power diagram, a 2D plane or area can be partitioned into non-empty cells as in Equation (9),

(9) $$P\left(S\right)=\left\{P\left(s_{i}\right)\vee i=1,\ldots ,n,P\left(s_{i}\right)\neq \emptyset \right\}.$$

**Figure 3:**
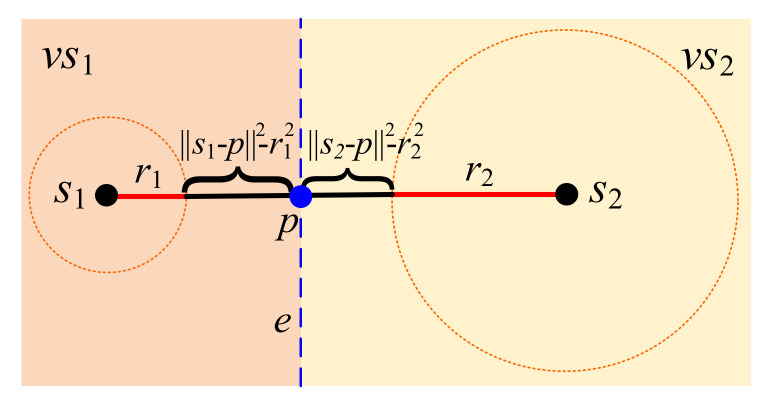
Example for power distances and power Voronoi cells in a power diagram.

As in Figure 3, an example demonstrates a power diagram with two sensors *s*_1_ and *s*_2_ with the respective weighted ranges *r*_1_ and *r*_2_. Here, there are two corresponding Voronoi cell *vs*_1_ and *vs*_2_.

In order to gain insight of the power diagram, let’s consider a simple power diagram formed by two sensors *s*_1_ and *s*_2_ with the respective weighted ranges *r*_1_ and *r*_2_ as in Figure 3. There are two corresponding Voronoi cells, *vs*_1_ and *vs*_2_. By setting or adjusting the weights of power diagram, the boundary, edge *e*, between two neighboring Voronoi cells *vs*_1_ and *vs*_2_ is close to one sensor with less residual energy as in Figure 3. In terms of path planning concerned, the mobile charging robot can drive through this edge, and then wirelessly charge and send/receive information for these two sensors *s*_1_ and *s*_2_, simultaneously. As a result, only one path is needed for balancing energy recharging and information transmission.

Thus, according to the power diagram, the weight can be set as $w_{i}=\frac{eng_{i}}{eng_{full}}$, thus the weighted range *r_i_* for each Voronoi cell $vc_{i}$ in the power Voronoi diagram becomes $r_{i}=r\times \frac{eng_{i}}{eng_{full}}$, where *eng_full_* is the full energy of sensor, and *eng_i_* is the residual energy of the sensor *s_i_*, 1 ≤ *i* ≤ *n*.

**Figure 4:**
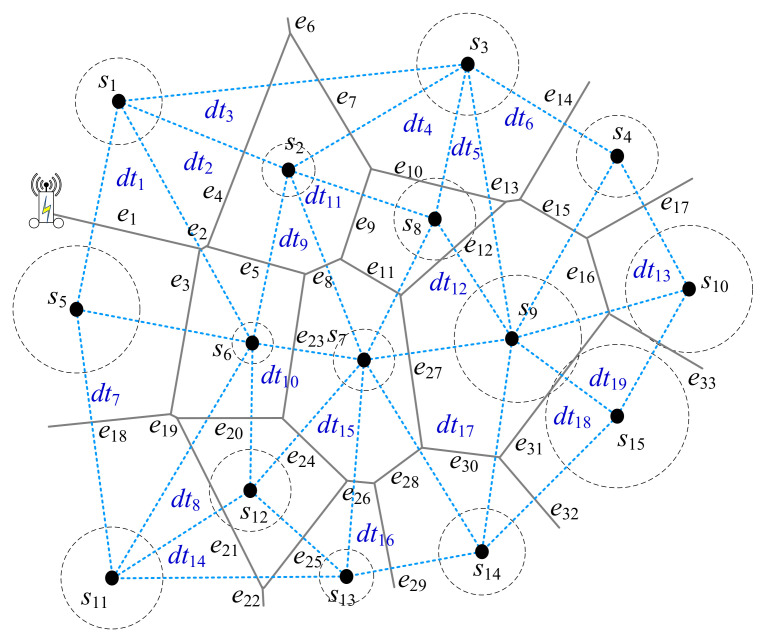
An example of power Voronoi diagram and its corresponding Delaunay triangulation.

The power Voronoi diagram is defined as a graph $G_{PD}=\left(V_{PD},E_{PD}\right)$. Delaunay triangulation and Voronoi diagram are dual in graph theory. The constructed Delaunay triangulation graph is defined as a graph $G_{DT}=\left(V_{DT},E_{DT}\right)$, where $V_{DT}=S$. An example with 15 sensor points *s_i_*, 1 ≤ *i* ≤ 15, in 2-D plane shows the graphs of power diagram and Delaunay triangulation as in Figure 4. Here, there are 33 edges *e_i_*, 1 ≤ *i* ≤ 33, as the boundary edges for power diagram with 15 Voronoi power cells $vc_{i}$, 1 ≤ *i* ≤ 15. *E_PD_* refers to as the set of these boundary edges of power Voronoi diagram. Here $E_{PD}=\left\{e_{1},e_{2},\ldots ,e_{33}\right\}$. In addition, there are 19 Delaunay triangulation $dt_{i}$, 1≤i≤19.

The main idea is described below. Suppose there are three sensors *s*_1_, *s*_5_, and *s*_6_ to form a Delaunay triangulation $dt_{1}$ as in Figure 4. In $dt_{1}$, there exist three edges *e*_1_, *e*_2_, and *e*_3_. If the *MR* is travelling from *e*_1_ to *e*_2_, from *e*_3_ to *e*_2_, or from *e*_1_ to *e*_3_, and vice versa, the three sensors *s*_1_, *s*_5_, and *s*_6_ can be charged by *MR* and their sensory data can be sent to *MR*. The scheduling for all of Delaunay triangulation $dt_{i}$, 1≤i≤19, visited once is important for *MR* with minimum power consumption. Based on the scheduling, all of sensors are charged by *MR* and all of their sensory data can be sent to *MR*. Thus, a path planning algorithm, named DT strategy, described below is proposed based on Delaunay’s triangulation graph as in Algorithm 1.
**Algorithm 1**: Path planning algorithm for mobile charging robot based on Delaunay triangulation.**INPUT**: *S* and *E_PD_*//S: the set of sensors, *E_PD_*: the set of boundary edges of power Voronoi diagram.**OUTPUT**: *P_DT_*//Scheduled traversal path based on DT strategy1.*P_DT_* = ∅2.Find the starting Delaunay triangulation *dt* with 3 Voronoi diagram edges *e_a_*, *e_b_*, *e_c_*, inside nearest to the *MR*.3.**While** (There exists one sensor marked UNCHARGED)4.{5. Suppose *MR* is on *e_a_* and *e_a_* is selected and appended into *P_DP_*. Both side sensors on *e_a_* are marked CHARGED.6. **If** (*f_uncharged_*(*e_b_*) > *f_uncharged_*(*e_c_*))7.  Both side sensors on *e_b_* are marked CHARGED.8.  *e_a_* ← *e_b_*9. **Else If** (*f_uncharged_*(*e_b_*) < *f_uncharged_*(*e_c_*))10.  Both side sensors on *e_c_* are marked CHARGED.11.  *e_a_* ← *e_c_*12. **Else If** (*f_uncharged_* (*e_b_*) == 0 and *f_uncharged_*(*e_c_*) == 0)13.  Find the nearest *dt*’ with 3 Voronoi diagram edges *e_a_*’, *e_b_*’, *e_c_*’ inside.14.  Suppose *e_a_*’ is nearest to *MR* and *MR* is moving to the nearest end point of *e_a_*’ constituted a path segment called *e_p_*, travelled unnecessary charging.15.  *e_p_* is appended into *P_DT_*.16.  *e_a_* ← *e_a_*’17. **Else**18.  $e_{m}=\underset{e_{b},e_{c}}{arg\;}\underset{i=b,c}{min\;}len\left(e_{i}\right)$ is appended into *P_DT_*19.  Both side sensors on *e_m_* are marked CHARGED.20.  *e_a_* ← *e_m_*21.}22.**Return***P*_*DP*_

CHARGED and UNCHARGED are Boolean variables which indicate whether the sensor is charged or uncharged, respectively, at this round for path scheduling. Assume all sensors are marked UNCHARGED at the beginning of Algorithm 1. The function *f_uncharged_*(*e*) is defined as the number of sensors on both sides of Voronoi diagram edge *e* marked UNCHARGED. The function *len*(*e*) is defined as the length of the edge *e*.

In the following, the time complexity of Algorithm 1 is discussed. Here the time complexities of finding the Voronoi diagram and Delaunay triangulation are all in *O*(*n* log_2_ *n*), where *n* is the number of sensors. The number of Voronoi cells is *n*. The number of triangles in Delaunay triangulation is in *O*(*n*). The time complexity of Step 1 is in *O*(1). Step 2 takes the time complexity *O*(*n*). The looping from Steps 3 to 21 is at most in *O*(*n*). Steps 5 to 20 takes in *O*(1) except that Step 13 takes in *O*(*n*) for finding the nearest *dt*’. Step 22 is in *O*(*n*). Therefore, the total time complexity of Algorithm 1 is in polynomial time complexity *O*(*n*^2^).

**Figure 5:**
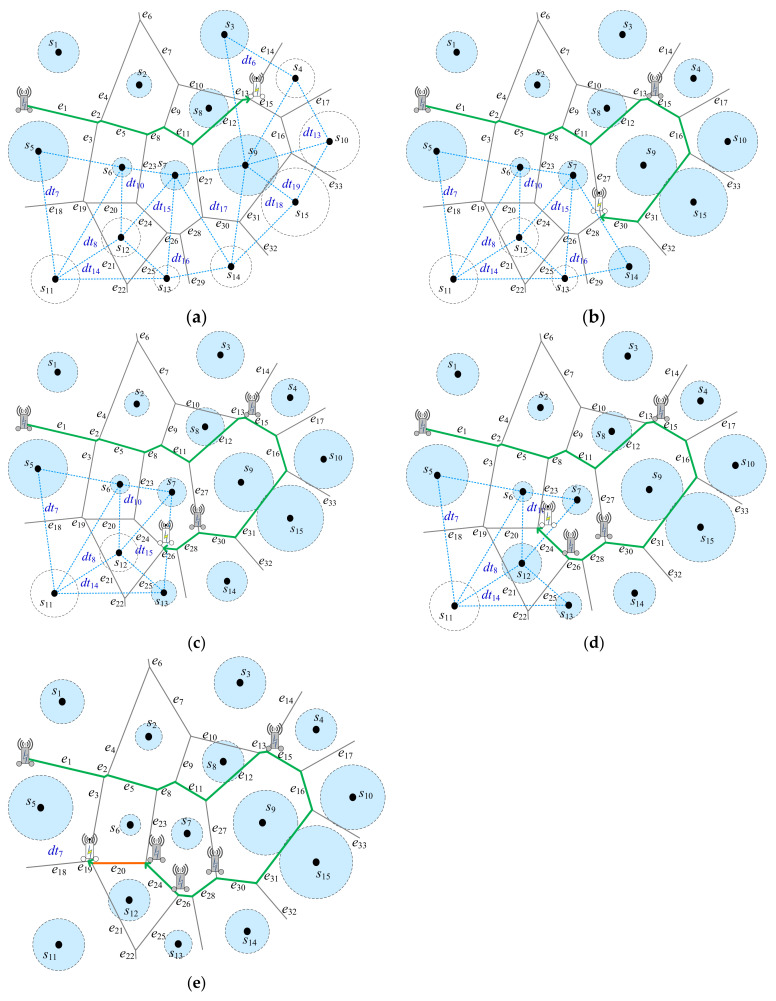
An example for DT strategy by Algorithm 1. (**a**) Route selection from orginal point, (**b**) Route planning in Step 17 after (**a**), (**c**) Route planning in Step 12 after (**b**), (**d**) Route planning in Step 12 after (**c**), (**e**) Route planning in Step 12 after (**d**).

**
Example** **1.
**
*Below an example in*Figure 4*will be demonstrated by Algorithm 1*. Figure 5*will show the detailed steps by Algorithm 1*. $S=\left\{s_{1},\ldots ,s_{15}\right\}$*and $E_{PD}=\left\{e_{1},\ldots ,e_{33}\right\}$. Initially, $P_{DT}=\emptyset$, at the beginning, all of sensors are marked UNCHARGED. MR is nearby the triangle $dt_{1}$, which is uncharged, as shown in Figure 4. Thus, $e_{a}=e_{1}$, $e_{b}=e_{2}$, and $e_{c}=e_{3}$ in $dt_{1}$. In Step 5, $e_{a}=e_{1}$ is appended into $P_{DT}$; $P_{DT}=\left\{e_{1}\right\}$. Sensors *s*_1_ and *s*_5_ are marked CHARGED. $f_{uncharged}$ ($e_{b}$) = $f_{uncharged}$($e_{c}$) = 1. It means that there are one sensor available for recharging. Next, $e_{m}=e_{b}=e_{2}$ is selected when $len\left(e_{2}\right)\leq len\left(e_{3}\right)$ as in*Figure 5a. *It means that the shorter moving distance is better while recharging the same number of uncharged sensors. Sensor *s*_6_ is marked CHARGED. $e_{m}$ is set to. Then, the while-loop is going to the next triangle $dt_{2}$. Here, $e_{a}=e_{2}$, $e_{b}=e_{4}$, and $e_{c}=e_{5}$ in $dt_{2}$. In Step 5, $e_{a}=e_{2}$ is appended into $P_{DT}$; $P_{DT}=\left\{e_{1},e_{2}\right\}$. Repeat the same steps. As a result, $P_{DT}=\left\{e_{1},e_{2},e_{5},e_{8},e_{11},e_{12},e_{13}\right\}$ obtained as shown in*Figure 5a. *Sensors *s*_1_, *s*_2_, *s*_3_, *s*_5_, *s*_6_, *s*_7_, *s*_8_, and *s*_9_ with light blue cycle around are marked CHARGED. Then, the same situations are repeatedly executed. As a result, $e_{15},e_{16},e_{31},e_{30}$ are appended into *P_DT_*; thus, $P_{DT}=\left\{e_{1},e_{2},e_{5},e_{8},e_{11},e_{12},e_{13},e_{15},e_{16},e_{31},e_{30}\right\}$ in*Figure 5b. *Sensors *s*_4_, *s*_10_, *s*_15_, and *s*_14_ with light blue cycle around are marked CHARGED. Next, *f_uncharged_*(*e*_27_) = *f_uncharged_*(*e*_28_) = 0 in Step 12. It means that there are no any sensor available for recharging. The nearest triangle $dt^{’}=dt_{16}$ is selected; thus, $e_{a}^{’}=e_{28}$, $e_{b}^{’}=e_{26}$, and $e_{c}^{’}=e_{29}$. Then, the same situations are repeatedly executed. As a result, $e_{28},e_{26}$ are appended into $P_{DT}$ as in*Figure 5c. *Next, $e_{m}=e_{b}=e_{24}$ is selected when $len\left(e_{24}\right)\leq len\left(e_{25}\right)$ as in*Figure 5d. *As a result, $e_{24}$ is appended into $P_{DT}$. Currently, sensor $s_{11}$ is not marked CHARGED. The triangle $dt^{’}=dt_{7}$ is selected. In Step 14, $e_{20}$ travelled only, no charging here, with orange color is appended into $P_{DT}$ as in*Figure 5e. *Next, $e_{19}$ is appended into $P_{DT}$. As a consequence, $P_{DT}=\left\{e_{1},e_{2},e_{5},e_{8},e_{11},e_{12},e_{13},e_{15},e_{16},e_{31},e_{30},e_{28},e_{26},e_{24},e_{20},e_{19}\right\}$. The green edges in $P_{DT}$ are needed to wirelessly recharge the sensors, whereas the orange edge is doing nothing, only MR moving along the orange edge*.

### 4.2. Path Planning Strategy Based on Dominating Set

In this subsection, a path planning strategy will be proposed based on the power Voronoi cells of sensors in a power Voronoi diagram.

This main idea of this path planning strategy is described below. At first, the power Voronoi diagram based on the residual energies for different sensors is established as described in Section 4.1. All of the power Voronoi cells of sensors in a power Voronoi diagram can be as nodes of the Delaunay triangulation graph. In the Delaunay triangulation graph, there exist edges among their neighboring Voronoi cells. In graph theory, based on a dominating set for the Delaunay triangulation graph $G_{DT}=\left(V_{DT},E_{DT}\right)$, it’s a subset *D* of $V_{DT}$ such that every vertex not in *D* is adjacent to at least one vertex of *D*. That is, if the *MR* is moving around the boundary of the power cell *vc* for a dominant sensor *s*, all of the sensors of its neighboring Voronoi cells including *s* can be charged and their corresponding sensory data can be received. Therefore, a path planning strategy will be proposed for *MR* scheduling to visit to all of dominants in *D*. As a result, all of sensors can be recharged by *MR* and all sensory data for sensors can be received by *MR*.

The proposed heuristic path planning algorithm for mobile charging robot based on Voronoi cell dominating set (for short VD strategy) is described in Algorithm 2. In the beginning, suppose *MR* is located in one Voronoi cell *vc*. Next, the strategy for finding the all dominants of a dominating set of $V_{DT}$ can be extended to finding these dominants with size *k* ≥ 1. That is, *k*-contiguous neighboring Voronoi cells can be condensed as one vertex. Moreover, if the *MR* is moving around the boundary of the *k*-contiguous neighboring Voronoi cells for a dominant with size *k*, all of the sensors of its neighboring Voronoi cells, including *k* sensors in the selected dominant can be charged and their corresponding sensory data can be received. $S_{VC}$($S_{VD}^{k}$) is defined as the set of Voronoi cells surrounded by *k*-contiguous neighboring Voronoi cells formed by a set of $S_{VD}^{k}$, where $\left|S_{VD}^{k}\right|=k$. $\left|S_{VC}\left(S_{VD}^{k}\right)\right|$ is denoted as the number of the set of Voronoi cells surrounding by *k*-contiguous neighboring Voronoi cells formed by a set of $S_{VD}^{k}$. In general, *k* is a constant and small; here, 1 ≤ *k* ≤ 3. Consequently, Algorithm 2 is proposed for planning *MR* to visit the boundary of all of these dominants with size *k* selected, named VD strategy.
**Algorithm 2**: Path planning algorithm for mobile charging robot based on Voronoi cell dominating set.**INPUT**: *S* and *E_PD_*, and *k* ≥ 3**OUTPUT**: *P_VD_*//Scheduled traversal path based on VD strategy1.*P_VD_* = ∅2.**While** (There exists one sensor marked UNCHARGED)3.{4. Suppose *MR* is located at the nearest Voronoi cell *vc* and its corresponding sensor *s_c_* is marked UNCHARGED.5. Find $S_{VD}^{k}$ with the maximum number of ∣ *S_vc_*($S_{VD}^{k}$)∣ with Voronoi cells marked UNCHARGED such that *vc* ∈ *S_vc_*($S_{VD}^{k}$) ∪ $S_{VD}^{k}$. If $S_{VD}^{k}$ is not found, decrease *k* by 1 and repeat Step 5.6.  Suppose that *k* Voronoi cells in $S_{VD}^{k}$ are surrounding by *q* edges *e*_1_, *e_2_*, …, *e_q_* in sequence in clockwise order. One end point *p* of *e*_1_ while *f_uncharged_*(*e*_1_) ≠ 0 is nearest to *MR*. *MR* moves *p* constituted a path segment called *e_s_*, travelled unnecessary charging. *e_s_*, *e*_1_, *e*_2_, …, *e_j_* in sequence are appended into *P_VD_* while *f_uncharged_*(*e_j_*) ≠ 0 and for all *f_uncharged_*(*e_l_*) = 0, *j* + 1 ≤ *e_l_* ≤ *q*. A short-cut edge can be added to replace the boundary edges in the Voronoi diagram. All *vc_i_* in *S_vc_*($S_{VD}^{k}$) ∪ $S_{VD}^{k}$ are marked CHARGED.7.}8.**Return***P_VP_*

In the following, the time complexity of Algorithm 2 is discussed. Step 1 is in *O*(1). The looping from Steps 2 to 7 is at most in *O*(*n*). Step 4 is in *O*(*n*) for searching the Voronoi cell *vc*. In Step 5, *k* being a constant are set to 1, 2, and 3 in this paper. When *k* is set to 1, 2 and 3, the time complexities of finding $S_{VD}^{k}$ is at most in *O*($n^{2}$), *O*($n^{3}$), and *O*($n^{4}$), respectively. Step 6 takes in *O*(*q*), where *q* is bounded by *O*(*n*). Step 8 is in *O*(*n*). Therefore, the total time complexity of Algorithm 2 is in polynomial time complexities *O*($n^{3}$), *O*($n^{4}$), and *O*($n^{5}$) with *k* set to 1, 2, and 3, respectively. Basically, when larger *k*, Algorithm 2 costs much for the running time. Here, *k* is a small constant.

The below three examples will be demonstrated by Algorithm 2.

**Figure 6:**
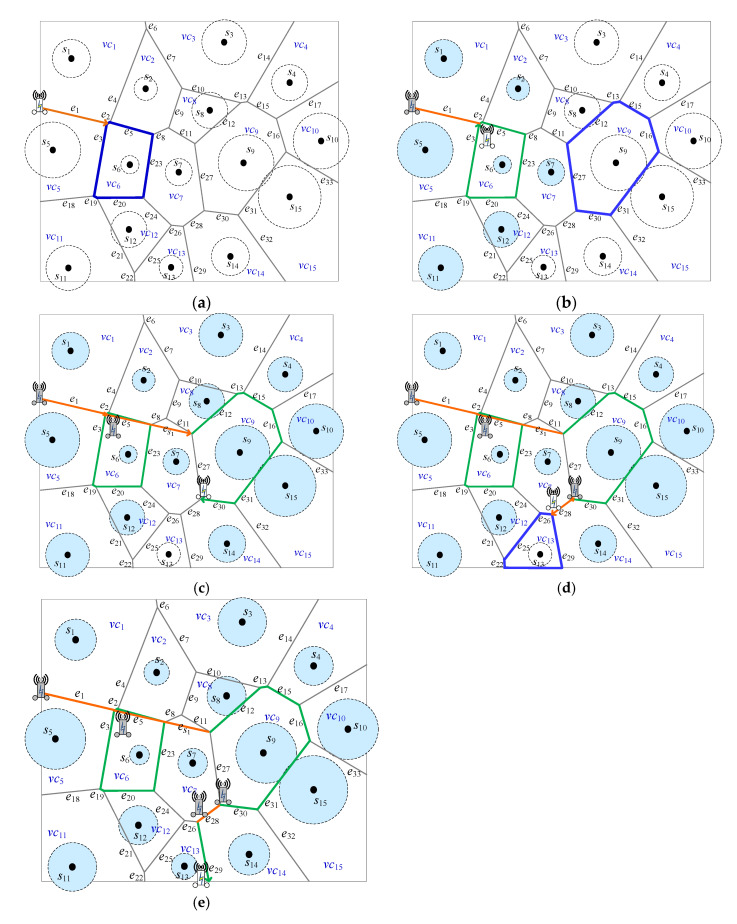
An example for path planning strategy based on dominating set with dominant size *k* = 1. (**a**) The first dominant selected set {*vc*_6_}, (**b**) The second selected dominant set {*vc*_9_} (**c**) Route planning after, (**b**), (**d**) The final selected dominant set *vc*_13_ (**e**) The final path planning for *MR*.

**
Example** **2.
**
Figure 6*shows the detailed steps of Algorithm 2 when k is set to 1. $S=\left\{s_{1},\ldots ,s_{15}\right\}$, $E_{PD}=\left\{e_{1},\ldots ,e_{33}\right\}$, and the set of Voronoi cells $S_{VC}=\left\{vc_{1},\ldots ,vc_{15}\right\}$. Initially, $P_{VD}=\emptyset$, at the beginning, all of sensors are marked UNCHARGED. In Step 4 of Algorithm 2, MR is nearby the Voronoi cells $vc_{1}$ and $vc_{5}$ based on the power distance, which is uncharged, as shown in*Figure 6a. *One of them will be selected; here suppose that $vc_{5}$ is selected and $s_{5}$ is marked UNCHARGED. In Step 5, the decision for four possibilities will be made as follows*.

1. Suppose $S_{VD_{1}}^{1}=\left\{vc_{1}\right\}$. Thus, $S_{VC}\left(S_{VD_{1}}^{1}\right)=\left\{vc_{3},vc_{2},vc_{6},vc_{5}\right\}$ where $vc_{5}\in S_{VC}$($S_{VD_{1}}^{1}$)$\cup S_{VD_{1}}^{1}$. $\left|S_{VC}\left(S_{VD_{1}}^{1}\right)\right|=4$; it means that there are four Voronoi cells surrounding the dominator $vc_{1}$ with size 1. In addition, in $S_{VC}\left(S_{VD_{1}}^{1}\right)$, the four corresponding sensors *s*_3_, *s*_2_, *s*_6_, and *s*_5_ are marked UNCHARGED, respectively.
2. Suppose $S_{VD_{2}}^{1}=\left\{vc_{6}\right\}$. Thus, $S_{VC}\left(S_{VD_{2}}^{1}\right)=\left\{vc_{1},vc_{2},vc_{7},vc_{12},vc_{11},vc_{5}\right\}$ where $vc_{5}\in S_{VC}$($S_{VD_{2}}^{1}$)$\cup S_{VD_{2}}^{1}$. $\left|S_{VC}\left(S_{VD_{2}}^{1}\right)\right|=6$; it means that there are six Voronoi cells surrounding the dominator $vc_{6}$ with size 1. In addition, in $S_{VC}\left(S_{VD_{2}}^{1}\right)$, the six corresponding sensors *s*_1_, *s*_2_, *s*_7_, *s*_12_, *s*_11_, and *s*_5_ are marked UNCHARGED, respectively.
3. Suppose $S_{VD_{3}}^{1}=\left\{vc_{11}\right\}$. Thus, $S_{VC}\left(S_{VD_{3}}^{1}\right)=\left\{vc_{5},vc_{6},vc_{12},vc_{13}\right\}$ where $vc_{5}\in S_{VC}$($S_{VD_{3}}^{1}$)$\cup S_{VD_{3}}^{1}$. $\left|S_{VC}\left(S_{VD_{3}}^{1}\right)\right|=4$; it means that there are four Voronoi cells surrounding the dominator $vc_{11}$ with size 1. In addition, in $S_{VC}\left(S_{VD_{3}}^{1}\right)$, the four corresponding sensors *s*_5_, *s*_6_, *s*_12_, and *s*_13_ are marked UNCHARGED, respectively.
4. Suppose $S_{VD_{4}}^{1}=\left\{vc_{5}\right\}$. Thus, $S_{VC}\left(S_{VD_{4}}^{1}\right)=\left\{vc_{1},vc_{6},vc_{11}\right\}$ where $vc_{5}\in S_{VC}$($S_{VD_{4}}^{1}$)$\cup S_{VD_{4}}^{1}$. $\left|S_{VC}\left(S_{VD_{4}}^{1}\right)\right|=3$; it means that there are three Voronoi cells surrounding the dominator $vc_{5}$ with size 1. In addition, in $S_{VC}\left(S_{VD_{4}}^{1}\right)$, the three corresponding sensors *s*_1_, *s*_6_, and *s*_11_ are marked UNCHARGED, respectively.

As in Figure 6b, in Step 5, $S_{VD_{2}}^{1}=\left\{vc_{6}\right\}$ is selected because $\left|S_{VC}\left(S_{VD_{2}}^{1}\right)\right|=6$ with Voronoi cells marked UNCHARGED is maximum number for the four possibilities as mentioned above. In Step 6, $P_{VD}=\left\{e_{1},e_{2},e_{5},e_{23},e_{20},e_{19},e_{3}\right\}$. Seven sensors *s*_1_, *s*_2_, *s*_7_, *s*_12_, *s*_11_, *s*_5_, and *s*_6_ are marked CHARGED.

After that, go to Step 2. There exist sensors marked UNCHARGED. Then, in Step 4, *MR* is near to the Voronoi cell $vc_{8}$, which, based on the power distance is uncharged, as shown in Figure 6b. In Step 5, the decision for many possibilities will be made. As a result, the $S_{VD}^{1}=\left\{vc_{9}\right\}$ is selected in Figure 6b. Thus, $S_{VC}\left(S_{VD}^{1}\right)=\left\{vc_{3},vc_{4},vc_{10},vc_{15},vc_{14},vc_{7},vc_{8}\right\}$ where $vc_{8}\in S_{VC}$($S_{VD}^{1}$)$\cup S_{VD}^{1}$. $\left|S_{VC}\left(S_{VD}^{1}\right)\right|=7$; it means that there are seven Voronoi cells surrounding the dominator $vc_{8}$ with size 1. In addition, in $S_{VC}\left(S_{VD}^{1}\right)$, there are six sensors *s*_3_, *s*_4_, *s*_10_, *s*_15_, *s*_14_, and *s*_8_ are marked UNCHARGED, respectively. In Step 6, one segment $e_{s_{1}}$ is built. Then, $P_{VD}=\left\{e_{1},e_{2},e_{5},e_{23},e_{20},e_{19},e_{3},e_{s_{1}},e_{12},e_{13},e_{15},e_{16},e_{31},e_{30}\right\}$ as shown in Figure 6c. Seven sensors *s*_8_, *s*_3_, *s*_4_, *s*_10_, *s*_15_, *s*_14_, and *s*_9_ are marked CHARGED. The segment of *e*_27_ is not appended into *P_VD_* because sensor *s*_7_ was marked CHARGED.

After that, repeat the same procedure and go to Step 2. There only exists one sensor *s*_13_ marked UNCHARGED. Then, in Step 4, *MR* is nearby the Voronoi cell $vc_{13}$ based on the power distance, which is uncharged, as shown in Figure 6c. In Step 5, the $S_{VD}^{1}=\left\{vc_{13}\right\}$ is selected. In Step 6, one segment $e_{s_{1}}=e_{28}$ is built in Figure 6d. Then, $P_{VD}=\left\{e_{1},e_{2},e_{5},e_{23},e_{20},e_{19},e_{3},e_{s_{1}},e_{12},e_{13},e_{15},e_{16},e_{31},e_{30},e_{28},e_{29}\right\}$ as shown in Figure 6e. *s*_13_ is marked CHARGED. Consequently, all of sensors were marked CHARGED and Algorithm 2 is done.

**Figure 7:**
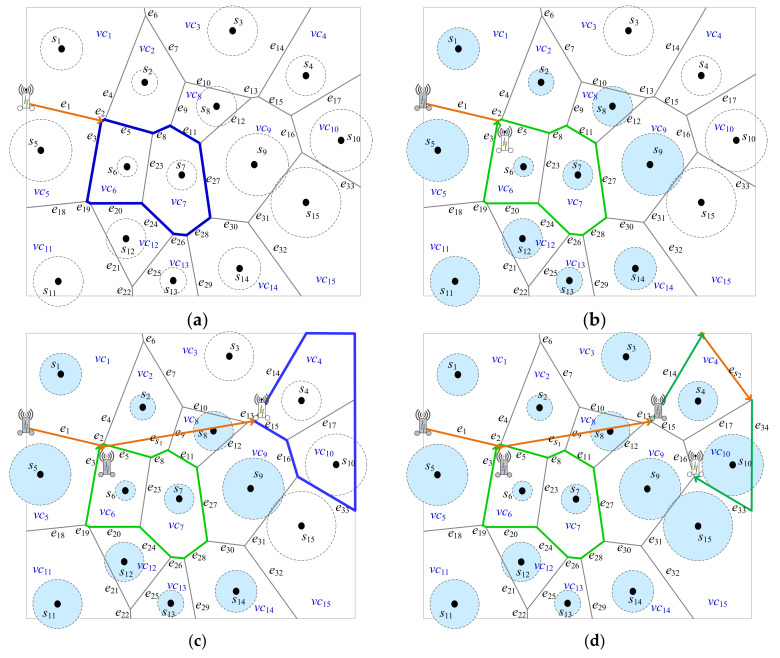
An example for path planning strategy based on dominating set with dominant size *k* = 2. (**a**) The first selected dominant set {*vc*_6_, *vc*_7_}, (**b**) Route planning after (**a**), (**c**) The final selected dominant set {*vc*_4_, *vc*_10_}, (**d**) The final path planning for *MR*.

**
Example** **3.
**
Figure 7*will show the detailed steps of Algorithm 2 when k is set to 2. $S=\left\{s_{1},\ldots ,s_{15}\right\}$, $E_{PD}=\left\{e_{1},\ldots ,e_{33}\right\}$, and the set of Voronoi cells $S_{VC}=\left\{vc_{1},\ldots ,vc_{15}\right\}$. At first, $P_{VD}=\emptyset$. At the beginning, all of sensors are marked UNCHARGED. In Step 4 of Algorithm 2, MR is nearby the Voronoi cells $vc_{1}$ and $vc_{5}$ based on the power distance, which is uncharged, as shown in*Figure 7 a. *As in Example 2, here suppose that $vc_{5}$ is selected and $s_{5}$ is marked UNCHARGED. In Step 5, the decision for many possibilities will be made. As a result, the $S_{VD}^{2}=\left\{vc_{6},vc_{7}\right\}$ is selected in* Figure 7a. $\left|S_{VC}\left(S_{VD}^{2}\right)\right|=9$ *with Voronoi cells marked UNCHARGED is maximum where $S_{VC}\left(S_{VD}^{2}\right)=\left\{vc_{1},vc_{2},vc_{8},vc_{9},vc_{14},vc_{13},vc_{12},vc_{11},vc_{5}\right\}$. It means that there are nine Voronoi cells surrounding the dominators $S_{VD}^{2}=\left\{vc_{6},vc_{7}\right\}$ with size 2. In Step 6, $P_{VD}=\left\{e_{1},e_{2},e_{5},e_{8},e_{27},e_{28},e_{26},e_{24},e_{20},e_{19},e_{3}\right\}$. Eleven sensors *s*_1_, *s*_2_, *s*_8_, *s*_9_, *s*_14_, *s*_13_, *s*_12_, *s*_11_, *s*_5_, *s*_6_, and *s*_7_ are marked CHARGED as shown in* Figure 7b.

After that, go to Step 2. There exist sensors marked UNCHARGED. Then, in Step 4, *MR* is nearby the Voronoi cell $vc_{3}$ based on the power distance, which is uncharged, as shown in Figure 7b. In Step 5, the decision for many possibilities will be made. As a result, the $S_{VD}^{2}=\left\{vc_{4},vc_{10}\right\}$ is selected in Figure 7c. Thus, $S_{VC}\left(S_{VD}^{2}\right)=\left\{vc_{15},vc_{9},vc_{3}\right\}$. $\left|S_{VC}\left(S_{VD}^{2}\right)\right|=3$; it means that there are three Voronoi cells surrounding the dominator $S_{VD}^{2}=\left\{vc_{4},vc_{10}\right\}$ with size 2. In addition, in $S_{VC}\left(S_{VD}^{2}\right)$, there are two sensors *s*_3_ and *s*_15_ are marked UNCHARGED. In Step 6, one segment $e_{s_{1}}$ is built as in Figure 7c. In addition, one short-cut $e_{s_{2}}$ is found as in Figure 7d. Consequently,$P_{VD}=\left\{e_{1},e_{2},e_{5},e_{8},e_{27},e_{28},e_{26},e_{24},e_{20},e_{19},e_{3},e_{s_{1}},e_{14},e_{s_{2}},e_{34},e_{33}\right\}$. Four sensors *s*_3_, *s*_4_, *s*_10_, and *s*_15_ are marked CHARGED. The segments of *e*_16_ and *e*_15_ are not appended into *P_VD_* because sensors *s*_9_ and *s*_8_ were marked CHARGED, respectively. Consequently, all of sensors were marked CHARGED and Algorithm 2 is done.

**Figure 8:**
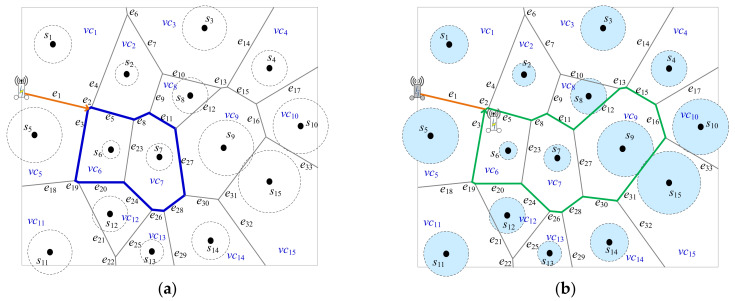
An example for path planning strategy based on dominating set with dominant *k* = 3. (**a**) The first selected dominant set {*vc*_6_, *vc*_7_, *vc*_9_}, (**b**) The final path planning for *MR*.

**
Example** **4.
**
Figure 8*will show the detailed steps step by step by Algorithm 2 when k is set to 3. $S=\left\{s_{1},\ldots ,s_{15}\right\}$, $E_{PD}=\left\{e_{1},\ldots ,e_{33}\right\}$, and the set of Voronoi cells $S_{VC}=\left\{vc_{1},\ldots ,vc_{15}\right\}$. Initially, $P_{VD}=\emptyset$, at the beginning, all of sensors are marked UNCHARGED. In Step 4 of Algorithm 2, MR is nearby the Voronoi cells $vc_{1}$ and $vc_{5}$, based on the power distance, which is uncharged, as shown in*Figure 8a *As in Example 2, suppose that $vc_{5}$ is selected and *s*_5_ is marked UNCHARGED. In Step 5, the decision for many possibilities will be made. As a result, the $S_{VD}^{3}=\left\{vc_{6},vc_{7},vc_{9}\right\}$ is selected in*Figure 8a. $\left|S_{VC}\left(S_{VD}^{3}\right)\right|=12$*with Voronoi cells marked UNCHARGED is maximum where $S_{VC}\left(S_{VD}^{3}\right)=\left\{vc_{1},vc_{2},vc_{8},vc_{3},vc_{4},vc_{10},vc_{15},vc_{14},vc_{13},vc_{12},vc_{11},vc_{5}\right\}$. It means that there are nine Voronoi cells surrounding the dominators $S_{VD}^{2}=\left\{vc_{6},vc_{7},vc_{9}\right\}$ with size 3. In Step 6, $P_{VD}=\left\{e_{1},e_{2},e_{5},e_{8},e_{11},e_{12},e_{13},e_{15},e_{16},e_{31},e_{30},e_{28},e_{26},e_{24},e_{20},e_{19},e_{3}\right\}$. Consequently, all of sensors were marked CHARGED and Algorithm 2 is done as shown in*Figure 8b.

### 4.3. Clusting for Mobile Charging Strategies

In this subsection, a cluster-based preprocessing for improving the charging strategies is presented. At first, anchors of clusters are selected in advanced for mobile charging strategies DT and VD. The selected anchors are representatives for those sensors which are nearest neighbors with each other. Basically, the presented approach is based on DBSCAN (Density-Based Spatial Clustering of Applications with Noise) clustering [36] which can be established to form clusters automatically. Initially, there are *n* rechargeable sensors *s_i_* in $S=\left\{s_{i}\left|i=1,2,\ldots ,n\right.\right\}$. Hereby, each cluster of sensors will be constrained with the range of two farthest sensors in the cluster. The range (or distance) will be called diameter of the cluster because mobile charger will efficiently charge the battery of each sensor in the cluster. Assume the value of $c_{diameter}^{max}$, the maximum diameter of cluster, is given. If the network is spare, *n* clusters are formed possibly whereas if the network is dense, only 1 cluster is formed possibly based on the $c_{diameter}^{max}$ setting.

Algorithm 3 is proposed for automatically clustering sensors and selecting an anchor location as the representative for each cluster. DBSCAN requires two parameters; *ε* is a certain radius of sensor and *minPts* is the minimum number of points required to form a dense region with radius *ε*. Here, initially, *minPts* is set to 1 adding to the maximum degree of the sensor in $\varepsilon \leq c_{diameter}^{max}$. Then, the clustering DBSCAN approach, named DB for short, repeatedly is performed after decreasing the value of *minPts* until to 2.
**Algorithm 3**: Clustering for sensors based on DBSCAN.**INPUT**: *S*, $c_{diameter}^{max}$, *ε* and *minPts***OUTPUT**: *S*’ and $S{’}_{Cluster}^{Anchor}$//*S*’: Clusters of sensors; $S{’}_{Cluster}^{Anchor}$: Anchors’ positions1.*minPts* = max (*deg*(*s_i_ in ε*) + 1)//(*deg*(*s_i_ in ε*) means the number of *s_i_* neighbors within *ε*.2.**For***minPts***downto** 23.{4. *S*’, $S{’}_{Cluster}^{Anchor}$ = Clustering (*S*, *ε*, *minPts*, $c_{diameter}^{max}$);5. If *S*’ ≡ *S*
**Then break**//Check sets equality of *S*’ and *S*6. *S* = *S*’ //Set *S*’ is assigned to *S*.7.}8.**Return***S*’ and $S{’}_{Cluster}^{Anchor}$**Procedure** Clustering (*S*, *ε*, *minPts*, $c_{diameter}^{max}$)1.*n_C_* = 0 //Cluster number2.**For** each point *S_p_* in *S*3.{4. **If** label (*S_p_*) ≠ **undefined Then Continue**5. *NB* = ∅6. **For** each point *S_q_* in *S* {7.  **If**
*dist*(*S_p_*, *S_q_*) ≤ *ε*
**Then** {*NB* = *NB* ∪ {*S_q_*} }8. **If** |*NB*| < *minPts*
**Then** { label(*S_p_*) = **Noise**; **Continue**;}9. *n_C_* = *n_C_* + 1; label(*S_p_*) = *n_C_*; *AS* = *NB*\{*S_p_*};10. **For** each point *S_q_* in *AS* {11.  **If** label(*S_q_*) = **Noise then** label(*S_q_*) = *n_C_*12.  **If** label(*S_q_*) ≠ **Undefined Then Continue**13.  label(*S_q_*) = *n_C_*; *NB* = ∅;14.  **For** each point *S_r_* in *S* {15.   **If**
*dist*(*S_q_*, *S_r_*) ≤ *ε*
**Then** {*NB* = *NB* ∪ {*S_r_*} }16.  **If** (|*NB*| ≥ *minPts* and $\max_{s_{1},s_{2}\in AS\cup NB}$
*dist*(*s*_1_, *s*_2_) ≤ $c_{diameter}^{max}$) **Then** {*AS* = *AS* ∪ *NB*}17.}18.*s_i_^c^* = {*s_j_*, 1 ≤ *j* ≤ *n*|*s_j_* is in cluster *i*}, 1 ≤ *i* ≤ *n_C_*19.*S_cluster_* = *S_i_^C^*|1 ≤ *i* ≤ *n_C_*20.$S_{Cluster}^{Anchor}$ = {*S_i_^CA^*|centroid (arithmetic mean position) of all the points in *S_i_^C^*}21.**Return***S_Cluster_* and $S_{Cluster}^{Anchor}$

The worst case of running time complexity remains *O*($n^{4}$) in Algorithm 3. Here, the size of $c_{diameter}^{max}$ will dominated to the clustering range. Therefore, *minPts* will be set to 1 adding to the maximum degree of the sensor in $\varepsilon \leq c_{diameter}^{max}$. *minPts* is decreased till to 2 to stop the execution, i.e., lastly, it will be set to be connected only with density size 2 in DBSCAN. The function label(*s_i_*)= $n_{C}$ is defined as labelling the clustering number $n_{C}$, $n_{C}\geq 1$, which each sensor node *s_i_* in $S=\left\{s_{i}\left|i=1,2,\ldots ,n\right.\right\}$ is belonging to.

Furthermore, applying the clustering strategy to DT and VT strategies will be discussed. First of all, the construction of power Voronoi diagram for clusters of sensors is needed. By Algorithm 3, the clusters *S*’ and anchors $S{’}_{Cluster}^{Anchor}$ of clusters are acquired. Here, each sensor position is replaced with the $s_{i}^{CA}$ for cluster $s_{i}^{C}$, $1\leq i\leq n_{C}$. As mentioned in Section 3, the weighting range *r_i_* for each Voronoi cell $vc_{i}$ in the power Voronoi diagram is set to $r_{i}=r\times \frac{eng_{i}}{eng_{full}}$, where *r* is constant, $eng_{full}$ is the full energy of sensor, and $eng_{i}$ is the average residual energy of the all sensors in $s_{i}^{C}$, $1\leq i\leq n_{C}$.

After that, the improvement algorithm with clustering can be constructed. When DBSCAN is adopted to DT is called DB-DT strategy, whereas DBSCAB is adopted to VD is called DB-VD strategy.

## 5. Experimental Results

In this section, experimental results are described. At first, the simulation environment and settings are presented in Section 5.1. Next, simulation results are presented in Section 5.2.

### 5.1. Simulation Environment

The designed simulator is implemented in JAVA programming language. The simulator is running on personal computer with Windows 10 operation system.

The parameter settings for simulation are shown as in Table 1. All of sensors are randomly deployed in the sensing field. In addition, the residual energy for each sensor is randomly assigned from 0 W to 5.3 W.

### 5.2. Simulation Results

The four proposed algorithms DT, VD, DB-DT, and DB-VD are studied based on greedy method GREEDY and clustering method CLUSTER, according to the following performance metrics:

- Total moving distance: including the total moving distance measured in meters for *MR* moving and charging at one round.
- Total energy consumption: including the energy consumptions measured in Joules for *MR* moving, *MR* charging for all sensors, and *MR* receiving sensory data from all sensors at one round.
- The completion time: including the total moving time measured in seconds for *MR* moving and charging at one round.

All of simulation results are obtained with the average values of 100 simulations. The GREEDY method is a *naïve* approach as a comparison baseline. In the beginning, in greedy method, a nearest sensor *s* is selected by *MR*. Then, *MR* is moving to the location of *s_a_* to charge its battery and receive its sensor data. While completed, an uncharged nearest sensor *s_b_* is selected by *MR*. *MR* is planned repeatedly with the same procedure while all of sensors are selected. CLUSTER method is applied by Algorithm 3 at first; after that, the traversal path is based on GREEDY method to complete the energy charging for *MR*. At the same time, CLUSTER method is as a comparison baseline for clustering-based methods. Here, 1-VD, 2-VD, and 3-VD stand for VD strategies with dominant sizes *k* = 1, 2, and 3, respectively. Hereby, a cell with a larger *k* ≥ 4 is not discussed because it may cause a sensor to be located inside more than three sensors; in the meanwhile, the sensors inside cannot be charged efficiently. Thus, performance comparisons are among GREEDY, DT, VD with 1-VD, 2-VD, and 3-VD approaches as well as CLUSTER, DB-DT, and DB-VD with DB-1-VD, DB-2-VD, and DB-3-VD approaches.

#### 5.2.1. Impact of *MR* Moving Total Distance

**Figure 9:**
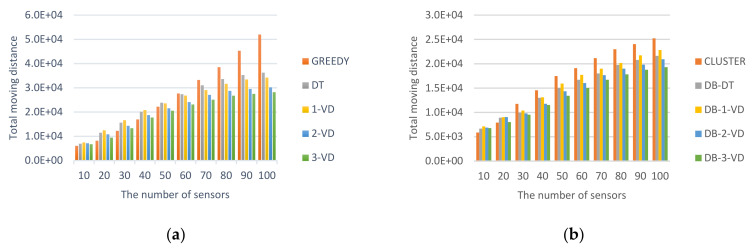
Total moving distance cost for *MR*. (**a**) Total moving distances for non-clustering strategies, (**b**) Total moving distances for clustering strategies.

Total moving distance for *MR* is a key factor to measure amount of energy and time saving can be achieved for all strategies. Figure 9 showed the performance comparisons for non-clustering and clustering-based strategies. For non-clustering strategies, they are required to consider all of sensors in one round. The total moving distances for constructed paths will be longer than those constructed in the clustering-based strategies will. For the impact factor, total moving distance, the clustering-based strategies have better efficiency.

At first, Figure 9a showed the total moving distances comparisons for GREEDY, DT, 1-VD, 2-VD, and 3-VD approaches without performing clustering. The distance is the total length of the path that represents the *MR* to visit all sensors to perform tasks around a round. For all approaches, as the number of sensors grows, the length of the *MR* traveled path becomes increased. Among them, GREEDY has a shorter path when the number of sensors is less than or equal 50; it represents a lower density and is more suitable for direct visits to the sensors, which will have a shorter total path length. But for the higher sensor density (the number of sensors is greater than or equal 50), GREEDY will have a worse performance and the path will become longer. Compared with 1-VD, DT will be better at the beginning. The main reason is that *MR* in DT will walk one travel pattern after another travel pattern. But when the density is higher, *MR* in DT may walk in random directions, resulting in not charged sensors partitioned to small blocks dispersed in the sensing field. This incurred that *MR* is necessary to travel a longer path to complete the charging task. This means that the higher the density, the better 1-VD will become. For the VD strategy, all of them are 1-VD, 2-VD and 3-VD. The larger the value of *k*, the shorter the total path lengths. As a result, the low density is very suitable for using GREEDY. The density is moderate, DT and VD are both very good. In high density, 3-VD will be the best candidate. 3-VD over GREEDY can acquire up to 45% improvement when *n* = 100 for *MR* moving distance.

Figure 9b showed the path comparisons for CLUSTER, DB-DT, DB-1-VD, DB-2-VD, and DB-3-VD approaches with performing clustering. For all clustering-based approaches, as the number of sensors increases, the length of the path *MR* travels becomes longer. Among them, the path is shorter in CLUSTER while the number of sensors is less than or equal 20. That is, while the density is lower, it is more suitable for direct access to the sensors, and there will be a shorter total path length. But, while density is higher (the number of sensors is greater than or equal 30), for CLUSTER the path will be longer. Compared with DB-1-VD, DB-DT will be better. The main reason is that *MR* in DB-DT will walk one traveled pattern after another traveled pattern. Compared with DB-2-VD, DB-DT will be better at the beginning. When the density is higher, *MR* in DB-DT may walk in random directions, causing non-charged sensors partitioned into small blocks dispersed in the sensing field. This incurred that *MR* is necessary to travel a longer path to complete the charging task. This means that the higher the density, the better DB-2-VD will become. For the DB-VD strategy, all of DB-1-VD, DB-2-VD and DB-3-VD are the larger the value of *k*, the shorter the total path lengths. As a result, the low density is very suitable for using CLUSTER. The density is moderate, both DB-DT and DB-VD are good among others. In high density, DB-3-VD will be the best choice. DB-3-VD over CLUSTER can acquire up to 23% improvement when *n* = 100 for *MR* moving distance.

#### 5.2.2. Impact of Energy Consumption

**Figure 10:**
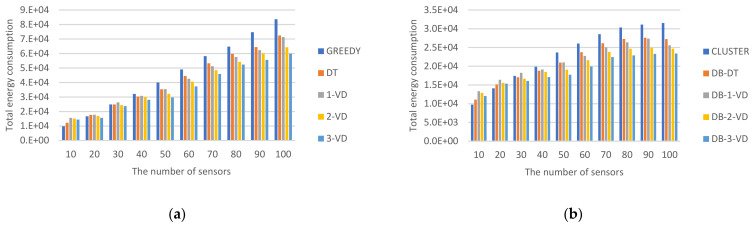
Total energy consumption cost for *MR*. (**a**) Energy consumption for non-clustering strategies, (**b**) Energy consumption for clustering strategies.

While *MR* moving to perform the task for charging sensors and acquiring the information from these sensors, energy consumption is the most concern for performance at a round. Doing this task is more consumed for the energy involved in power consumptions of *MR* moving for the designated path, *MR* charging sensors with different residual energy and retrieving sensory data from all of sensors. Figure 10 shows the total energy cost for recharging all of sensors in a round for different strategies.

At first, the energy consumption comparisons for GREEDY, DT, 1-VD, 2-VD, and 3-VD approaches without performing clustering are shown in Figure 10a. It is obvious that the increased of number of sensors, length of traversal paths, time of acquiring data from sensors will cause the higher energy consumed by the *MR*.

Among all, GREEDY has better energy consumption when the network size is relatively small. The main reason is that *MR* moves towards the sparse sensor, and then performs the task of collecting data and performing charging battery. Other strategies require charging and collecting data separately along both sides of a longer path. A longer radius of charging and collecting data, as well as a longer path for *MR*, cause *MR* to consume more energy, which is not conducive to dual-side charging. When the sensors are densely distributed and more sensors are in the sensing range, for the proposed approaches, *MR* charged both sides at the same time. Since the radius of collecting data become shorter, the walking path will be shorter. In an increasingly dense environment, the energy consumption to complete a round is better than the baseline GREEDY.

In addition, the energy consumption of DT is fair for small networks, namely below size of 10. DT has relatively good energy consumption. This is based on the fact that in DT *MR* tried to find a neighboring path that can charge and collect data at the same time. In VD, after *MR* traveled a VD with size 1, 2, or 3, *MR* then tried to walk the next VD with the same size. But when the network size becomes larger, it may cause excessive cutting of the network, causing multiple subnetworks to separate and the walking path, which gradually become longer. It will consume more power. For 1-VD, 2-VD, and 3-VD, as the value of *k* becomes larger, the more power-saving, the better the performance. Among them, in 3-VD, the size of local 3-VD is larger, more sensors are charged. 3-VD over GREEDY can acquire up to 28% improvement when *n* = 100 for *MR* energy consumption.

Next, the energy consumption comparisons for CLUSTER, DB-DT, DB-1-VD, DB-2-VD, and DB-3-VD approaches with performing clustering are shown in Figure 10b. All of total energy consumption are less than those without clustering, i.e., many sensors with recharging and information retrieved from *MR* visiting them together can save more energy. While the number of sensors increasing, energy consumptions are increased for each strategy with clustering as well. In the meanwhile, the more the lengths of traversal paths and the number of sensors with recharging and getting information requirements all of strategies were performed by *MR*, the higher energy will be consumed for *MR*.

Simulation performance and analysis for clustering-based and non-clustering approaches are similar. CLUSTER has better energy consumption when the network size is relatively small. The main reason is that *MR* moves towards the sparse sensor, and then performs the task of collecting data and performing charging battery. Other strategies require charging and collecting data separately along both sides of a longer path. A bigger radius of charging and collecting data, as well as a longer path for *MR*, cause *MR* to consume more energy, which is not conducive to dual-side charging. When the sensors are densely distributed and more sensors are in the sensing range, for the proposed approaches, *MR* charged both sides at the same time. This is because that the charging radius and the radius of collecting data become shorter and the walking path becomes shorter. In an increasingly dense environment, the energy consumption to complete a round is better than the baseline CLUSTER approach.

In addition, the energy consumption of DB-DT is good for the network below size of 20. This is based on the fact that, in DB-DT, *MR* tried to find a neighboring path that can charge and collect data at the same time every time. In DB-VD, after *MR* traveled a DB-VD with size 1, 2, or 3, *MR* then tried to walk the next VD with the same size. But when the network size becomes larger, it may cause excessive cutting of the network, causing multiple subnetworks to separate and the walking path, which gradually become longer. It will consume more power. For DB-1-VD, DB-2-VD, and DB-3-VD, as the value of *k* becomes larger, the more power-saving, the better the performance. In summary, for DB-3-VD, the size of local DB-3-VD is larger, more sensors are charged efficiently for *MR*. DB-3-VD over CLUSTER can acquire up to 25% improvement when *n* = 100 for *MR* energy consumption.

#### 5.2.3. Impact of Completion Time

**Figure 11:**
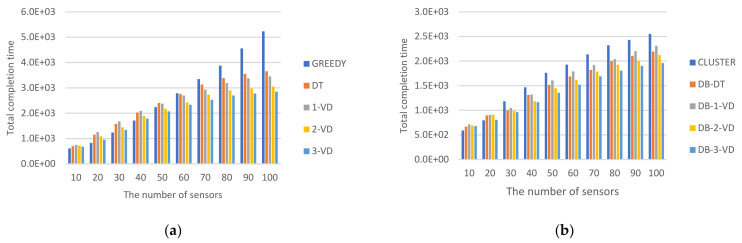
Total completion time spent for *MR.* (**a**) Total completion time for non-clustering strategies, (**b**) Total completion time for clustering strategies.

Total completion time for *MR* is a key factor to measure how much amount of time spent will be achieved for a strategy. Figure 11 showed the performance comparisons for non-clustering and clustering-based strategies. For non-clustering strategies required to consider all of the sensors in one round, the total completion time of *MR* for accomplishing the charging task will be longer than those in the clustering-based strategies. This is the advantage of the clustering-based strategies.

Figure 11a showed the total completion time comparisons for GREEDY, DT, 1-VD, 2-VD, and 3-VD approaches without performing clustering. The completion time is to accomplish the designated task in which *MR* visits all sensors to perform tasks around a round. For all approaches, as the number of sensors increases, the completion time of the *MR* travels path becomes longer. Among them, GREEDY has a shorter completion time when the number of sensors is less than or equal 50; it represents a lower density and is more suitable for direct visits to the sensors, which will have a shorter completion time for charging due to low charging distance. But for the higher sensor density, GREEDY will have a worse performance and the completion time will become longer. Compared with 1-VD, DT will be better at the beginning. The main reason is that *MR* in DT will walk one travel pattern after another travel pattern. But when the density is higher, *MR* in DT may walk in random directions, resulting in not charged sensors partitioned to small blocks dispersed in the sensing field. This incurred that *MR* is necessary to travel a longer path to complete the charging task. This means that the higher the density, the better 1-VD will become. For the VD strategy, all of them are 1-VD, 2-VD and 3-VD. The larger the value of *k*, the shorter the total completion time. As a result, the low density is very suitable for using GREEDY. The density is moderate, DT and VD are both very good. At high density, 3-VD will be the best. 3-VD over GREEDY can acquire up to 45% improvement when *n* = 100 for *MR* completion time.

Figure 11b showed the total completion time comparisons for CLUSTER, DB-DT, DB-1-VD, DB-2-VD, and DB-3-VD approaches with performing clustering. For all clustering-based approaches, as the number of sensors increases, the total completion time of *MR* travels becomes longer. Among them, the total completion time is shorter in CLUSTER while the number of sensors is less than or equal 20. That is, while the density is lower, it is more suitable for direct access to the sensors, and there will be a shorter total completion time. But, while density is higher (the number of sensors is greater than or equal 20), for CLUSTER the path will be longer. Compared with DB-1-VD, DB-DT will be better. Compared with DB-2-VD, DB-DT will be better at the beginning. The main reason is that *MR* in DB-DT will walk one travel pattern after another travel pattern. When the density is higher, *MR* in DB-DT may walk in random directions, causing non-charged sensors partitioned into small blocks dispersed in the sensing field. This incurred that *MR* is necessary to travel a longer path to complete the charging task. This means that the higher the density, the better DB-2-VD will become. For the DB-VD strategy, all of DB-1-VD, DB-2-VD and DB-3-VD are the larger the value of *k*, the shorter the total completion time. As a result, the low density is very suitable for using CLUSTER. The density is moderate; both DB-DT and DB-VD are very good. At high density, DB-3-VD will be the best. DB-3-VD over CLUSTER can acquire up to 23% improvement when *n* = 100 for *MR* completion time.

## 6. Conclusions

This paper proposed wireless dual-side charging strategies by mobile charging robots for wireless rechargeable sensor networks. At first, a power diagram was constructed according to the remaining power of sensors and distances among sensors in a WRSN. Based on this built power diagram, charging strategies for DT and VD were addressed with dual-side charging capability. In a high-density sensing network, they can effectively reduce the energy consumption, total distance traveled, and the time to complete the recharging. The dual-side charging capability for *MR* can balance the efficiency of recharging the sensors on both sides during mobile charging. In simulations, the performance for GREEDY, DT, 1-VD, 2-VD, 3-VD were analyzed and compared. DT has better efficiency in lower density WRSNs, whereas 3-VD has the best efficiency in higher density WRSNs. Furthermore, a clustering method, named DB, was proposed to group the neighboring sensors according to the considerations of their charging range and density. In terms of applying DB to DT and VD strategies, DB-DT and DB-VD were proposed, respectively. According to these two kinds of clustering strategies compared to the non-clustering strategies, the energy consumption, the walking distance, and the time to complete the charging can be greatly reduced. In simulations, the performance for CLUSTER, DB-DT, DB-1-VD, DB-2-VD, and DB-3-VD were analyzed and compared. DB-DT has better efficiency in lower-density WRSNs, while DB-3-VD has the best efficiency in high-density WRSNs. It can be seen that clustering has better efficiency than non-clustering. DT and DB-DT are suitable for low-density WRSNs, whereas 3-VD and DB-3-VD are suitable for high-density WRSNs. Consequently, all of the strategies proposed in this paper are suitable for different clustering technologies integrated into DT and VD. In short, dual-side clustering-based strategies will be possible to get better efficiency of energy and distance saving.

## Figures and Tables

**Table 1:** Simulation parameter settings.

Parameters	Values
Simulation area	1000 m × 1000 m
Antenna resistance	50 Ω
Origin location of *MR*	(0, 0) at the top left corner
Recharging power of *MR*	18 W
Maximum moving speed of *MR*	10 m/sec
Sensor energy capacity	5.3 W
Consumption of sensing	0.02 W
Recharging range of sensor	300 m
Communication range of sensor	300 m
*η*	4.32 × 10^−4^
*β*	30 m
Packet size	1024 bits
*ε*	30 m
$c_{diameter}^{max}$	50 m
The number of sensors (Network sizes (*n*))	10~100

## Data Availability

Not applicable.
